# A promising mRNA vaccine derived from the JN.1 spike protein confers protective immunity against multiple emerged Omicron variants

**DOI:** 10.1186/s43556-025-00258-7

**Published:** 2025-03-04

**Authors:** Danyi Ao, Dandan Peng, Cai He, Chunjun Ye, Weiqi Hong, Xiya Huang, Yishan Lu, Jie Shi, Yu Zhang, Jian Liu, Xiawei Wei, Yuquan Wei

**Affiliations:** 1 Laboratory of Aging Research and Cancer Drug Target, State Key Laboratory of Biotherapy and Cancer Center, National Clinical Research Center for Geriatrics, West China Hospital, Sichuan University, No. 17, Block 3, Southern Renmin Road, Chengdu, 610041 Sichuan China; 2 WestVac Biopharma Co. Ltd., Chengdu, China

**Keywords:** SARS-CoV-2, JN.1 variant, mRNA vaccine, Heterologous vaccination, Recombinant protein vaccine, Immune response

## Abstract

Despite the declared end of the COVID-19 pandemic, SARS-CoV-2 continues to evolve, with emerging JN.1-derived subvariants (e.g., KP.2, KP.3) compromising the efficacy of current XBB.1.5-based vaccines. To address this, we developed an mRNA vaccine encoding the full-length spike protein of JN.1, incorporating GSAS and 2P mutations and encapsulated in lipid nanoparticles (LNPs). The JN.1-mRNA vaccine elicited robust humoral and cellular immune responses in mice, including high JN.1-specific IgG titers, cross-neutralizing antibodies, and increased T follicular helper (Tfh) cells, germinal center (GC) B cells, and T cell cytokines. Importantly, immunity persisted for up to six months and induced RBD-specific long-lived plasma cells. We also compared the immune responses induced by homologous and heterologous vaccination regimens, and our results demonstrated that the heterologous regimen—combining JN.1-mRNA with a recombinant protein vaccine (RBD_JN.1_-HR)—induced stronger responses. These findings highlight the JN.1-mRNA vaccine constitutes an effective prophylactic approach against JN.1-related variants, as it induces potent neutralizing antibody responses across all tested lineages. This enhanced immunogenicity is expected to significantly reduce hospitalization rates and mitigate post-COVID complications associated with JN.1 and KP.3 infections. This study emphasizes the need for timely vaccine updates and the adaptability of mRNA vaccines in addressing emerging pathogens, providing a framework for combating future infectious diseases. Collectively, these results offer critical insights for vaccine design and public health strategies in response to emerging SARS-CoV-2 variants.

**Supplementary Information:**

The online version contains supplementary material available at 10.1186/s43556-025-00258-7.

## Introduction

The COVID-19 pandemic, caused by the SARS-CoV-2 virus, has significantly reshaped global social, economic, and healthcare landscapes. Although five years have passed since the outbreak, the virus’s impact continues to reverberate across the globe. Variants of SARS-CoV-2, such as the Delta and Omicron strains, have emerged over time, inducing different challenges to public health measures. These variants often exhibit enhanced transmissibility and immune evasion capabilities, leading to successive waves of infections even as vaccination campaigns progress. With emerging subvariants continuing to dominate, the global health community faces an ongoing need for adaptive strategies to combat these evolving threats, highlighting the necessity of continuous updates to vaccines and other public health interventions.

In late 2023, a mutant strain, JN.1, emerged and caused a significant surge in infections across the United States and Europe. Within a short period, JN.1 and its many descendants (KP.3, KP.3.1.1, XDV.1, LB.1, and XEC) supplanted XBB.1.5, EG.5, and other epidemic strains to become the globally predominant [1–3]. JN.1, a sublineage of BA.2.86, is characterized by the hallmark spike protein mutation L455S, which confers enhanced viral infectivity and immune evasion compared to other circulating variants [4]. While the current XBB.1.5 monovalent vaccine provides reasonable protection, the heightened neutralization resistance of JN.1 subvariants presents a growing concern [5]. In response, the World Health Organization (WHO) and the U.S. Food and Drug Administration (FDA) have called for the inclusion of JN.1 spike components in the development of future vaccines to improve efficacy against these evolving variants [6, 7].

mRNA technology has gained significant attention in modern vaccine development due to its rapid scalability, potent immune responses, and straightforward formulation [8]. The mRNA vaccine is a highly adaptable platform, enabling the creation of variant-specific vaccines by altering the antigenic sequence of the encoded protein [9–11]. This flexibility is critical for addressing rapidly mutating pathogens like SARS-CoV-2. Additionally, mRNA vaccines have a favorable safety profile, characterized by a short pharmacokinetic half-life, lack of genomic integration, and reduced CpG content, which make them safer compared to viral vector-based or DNA vaccines [12, 13]. Moreover, mRNA vaccines effectively stimulate robust humoral and cellular immune responses to provide biological interactions to defend the host against the virus, especially inducing strong Th1-immune responses [14–16], thereby further enhancing their efficacy [17–19]. These characteristics underscore the potential of mRNA technology as a versatile and effective platform for addressing both current and future infectious disease threats.

Homologous immunity, which refers to the immune response generated by repeated exposure to the same antigen or vaccine platform, and heterologous immunity, which involves the immune response elicited by different antigens or vaccine platforms. Within this context, heterologous vaccination strategies, which combine distinct vaccine platforms for priming and boosting, have demonstrated significant advantages in enhancing both the magnitude and breadth of immune responses compared to homologous vaccination approaches [20–22]. The synergistic enhancement of both humoral and cellular immune responses supports heterologous vaccination as a robust strategy for eliciting broad-spectrum protection against SARS-CoV-2 and its emerging variants.

In this study, we have introduced a novel approach by combining mRNA and recombinant protein vaccine platforms to combat emerging variants. We developed the JN.1-mRNA vaccine, which encodes the full-length spike protein of the JN.1 variant, and demonstrated its ability to generate long-lasting humoral and cellular immune responses in mice, with effects lasting up to 6 months. Additionally, we developed the RBD_JN.1_-HR recombinant protein vaccine based on the receptor-binding domain of the JN.1 variant [23]. Most importantly, our study revealed the groundbreaking efficacy of heterologous prime-boost immunization, where the combination of the JN.1-mRNA vaccine followed by a booster dose of the RBD_JN.1_-HR protein vaccine induced significantly stronger immune responses than homologous immunization regimens. This novel prime-boost strategy highlights a promising new direction for vaccine development, offering enhanced protection and flexibility in addressing rapidly evolving viral variants.

## Results

### JN.1-mRNA vaccine preparation and characterization

**Fig. 1:**
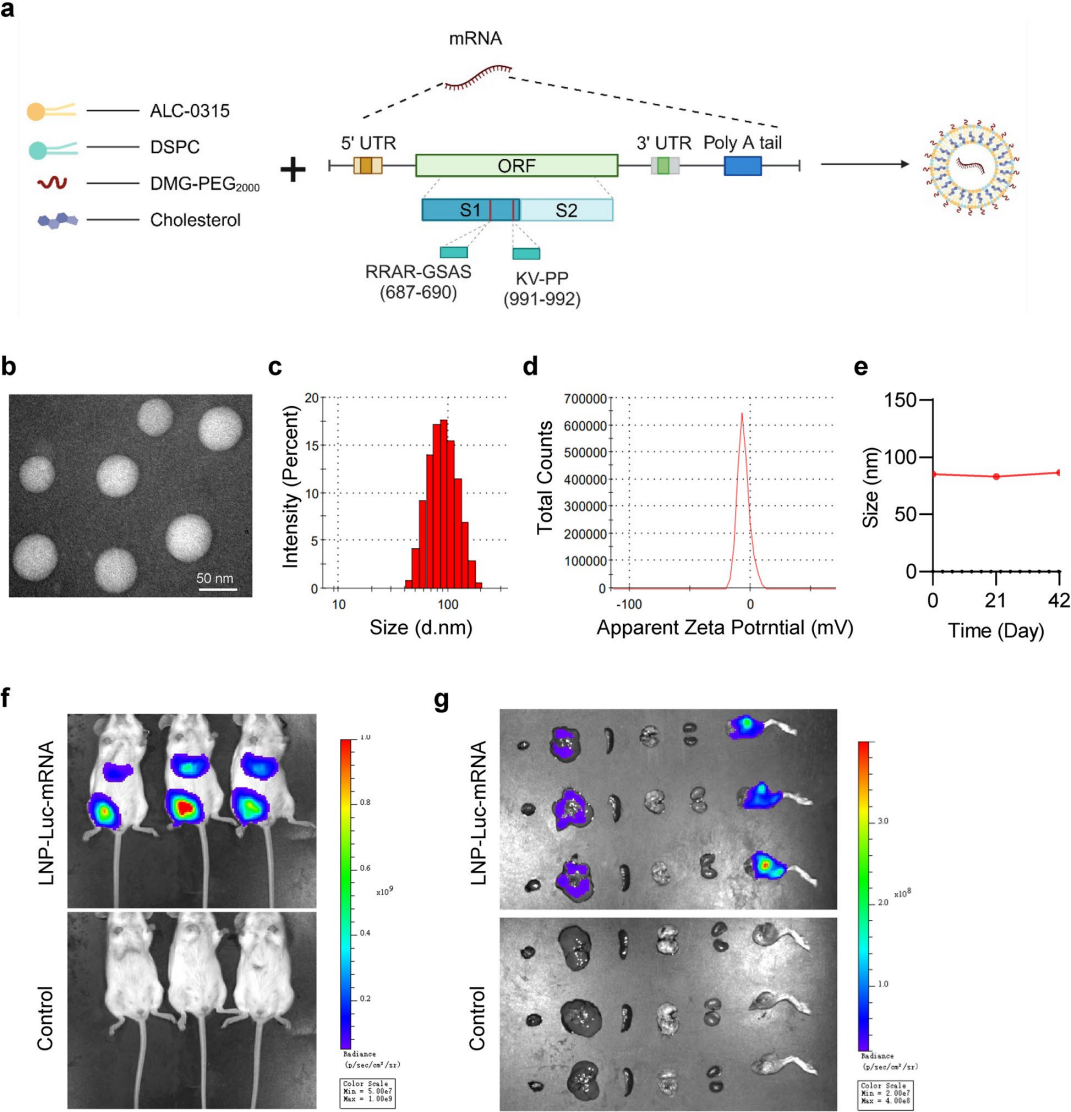
JN.1 mRNA-LNP vaccine construction, production and characterization. **a** Scheme of prefusion-stabilized JN.1 variant mRNA construct design. **b** Transmission Electron Microscopy (TEM) image of JN.1-mRNA vaccine. Scale bars, 50 nm. **c**-**d** The particle size distribution and zeta potential of JN.1-mRNA-LNP. **e** Changes in particle size of JN.1-mRNA-LNP after 42 days of storage at −80 °C. **f**-**g** Images of in vivo and ex vivo organs bioluminescence after 6 h injected 20 µg Luc-mRNA formulation, showing the distribution of the injected mRNA-LNP (*n* = 3 per group)

To construct the JN.1-mRNA vaccine, we designed a construct with a 5′ untranslated region (UTR), an open reading frame (ORF), a 3′ UTR, and a poly(A) tail (Fig. 1a). The ORF encodes the full-length spike protein of the JN.1 variant, incorporating a pre-fusion stabilized mutation (2P) and a furin cleavage-site mutation (GSAS) in the S1 domain [24]. The spike mRNA was mixed with ALC0315 through a microfluidics device to produce the mRNA-LNP formulation (Fig. 1a). Transmission electron microscopy analysis revealed that the JN.1-mRNA vaccine exhibits a spherical shape with a homogeneous size (Fig. 1b). The JN.1-mRNA vaccine exhibited a monodispersed size distribution with an average particle diameter of 85.02 ± 0.7 nm, and a zeta potential of −7.34 ± 1.48 mV (Fig. 1c, d). The particle size of the JN.1-mRNA vaccine remained stable at approximately 85 nm over a storage period of 42 days at 4 ℃, highlighting its physical stability (Fig. 1e). To evaluate the in vivo distribution and expression of the vaccine, luciferin (Luc)-mRNA was administered intramuscularly into BALB/c mice, while phosphate-buffered saline (PBS) served as the negative control. Six hours post-administration, images of in vivo and ex vivo organs showed that Luc-mRNA expression was predominantly localized at the injection site, with less expression observed in the liver (Fig. 1f, g).

### Robust immune responses of the JN.1-mRNA vaccine

**Fig. 2:**
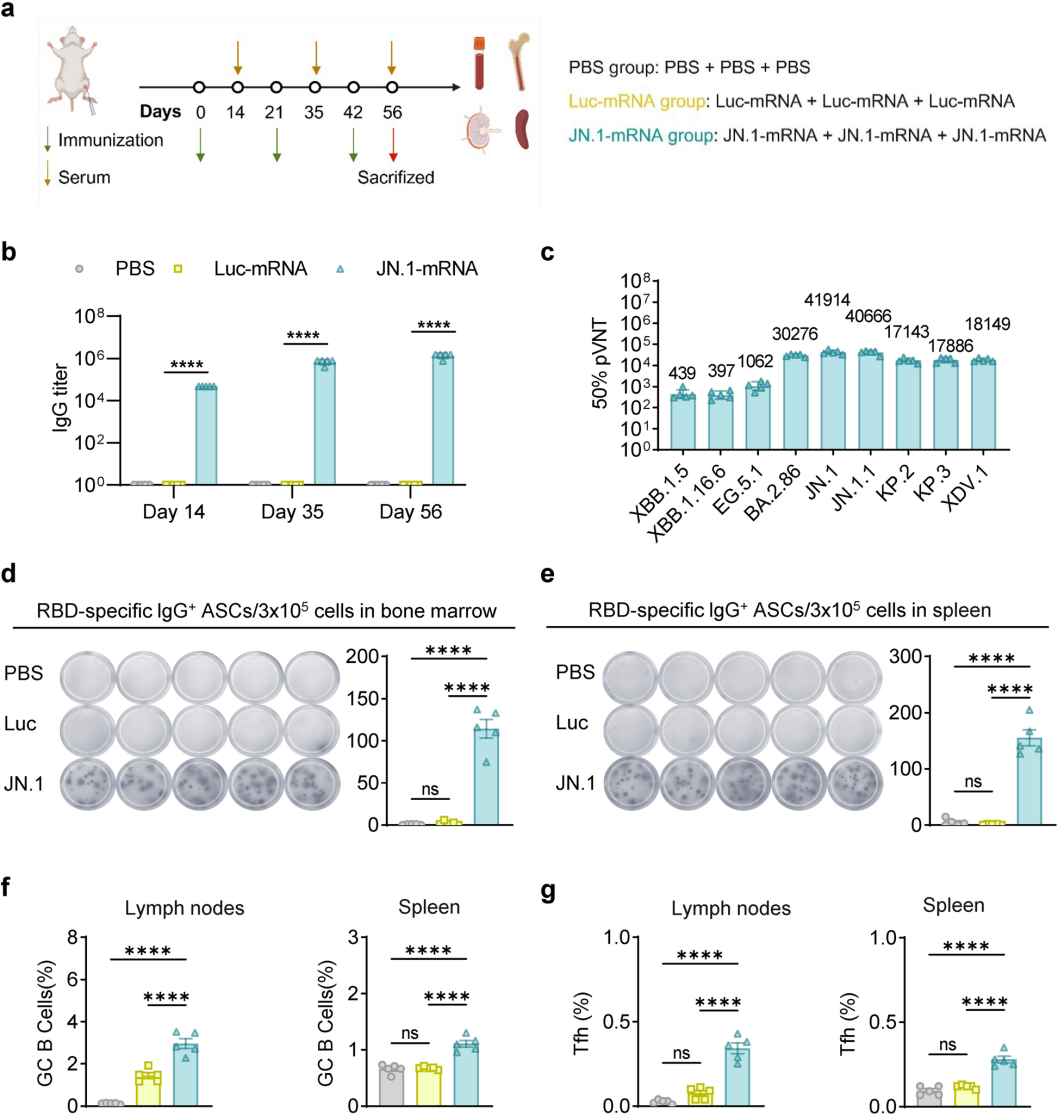
The JN.1-mRNA vaccine elicited robust humoral immune responses. **a** Experimental timeline for immune and tissue sample collection, along with experimental grouping (*n* = 5). **b** The RBD-specific binding antibody titers in serum after immunization. **c** Neutralizing antibodies in serum 14 d post-immunization. **d**-**e** ELISpot images (left) and spot counts (right) showing RBD-specific IgG^+^ ASCs in bone marrow (**d**) and spleen (**e**) of vaccinated mice. **f**-**g** The percentage of GC B cells (**f**) and Tfh cells (**g**) within lymph nodes (left) and spleen (right) 14 d after the third vaccination. All means were compared to each other with one-way ANOVA. *P < 0.05 was considered statistically significant. **P < 0.01 and ***P < 0.001 were considered highly significant

**Fig. 3:**
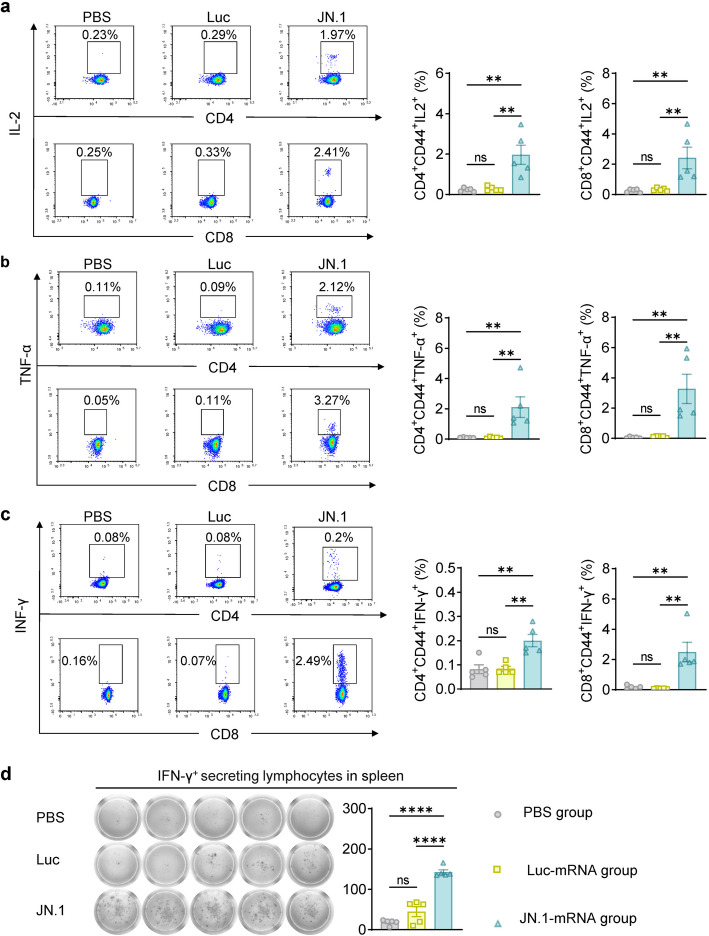
The JN.1-mRNA vaccine elicited robust cellular immune responses. **a**-**c** Percentages of IL-2-, TNF-α and IFN-γ-positive cells in CD4^+^ and CD8^+^ T cells from spleen. **d** ELISpot images (left) and spot counts (right) of IFN-γ-secreting T cells in the spleen. Experimental group *n* = 5. All means were compared to each other with one-way ANOVA. *P < 0.05 was considered statistically significant. **P < 0.01 and ***P < 0.001 were considered highly significant

To assess the immunogenicity of the JN.1-mRNA vaccine, 6–8-week-old BALB/c mice were immunized with three doses of JN.1-mRNA vaccine (10 μg per dose) on days 0, 21, and 42. Control groups received either PBS or Luc-mRNA under the same conditions. Serum samples were collected on days 14, 35, and 56 to evaluate binding and neutralizing antibody responses. Additionally, lymphocytes were harvested from the bone marrow, inguinal lymph nodes, and spleens on day 56 for further analysis (Fig. 2a). A single dose of the JN.1-mRNA vaccine effectively elicited robust titers of RBD-specific IgG (Fig. 2b). The pseudovirus neutralization test (pVNT) showed that the three-dose regimen induced high titers of neutralizing antibodies against XBB.1.5, XBB.1.16.6, EG.5.1, BA.2.86, JN.1, JN.1.1, KP.2, KP.3 and XDV.1 pseudoviruses, with geometric mean titers (GMTs) for 50% neutralization at 439, 397, 1062, 30,276, 41,914, 40,666, 17,143, 17,886, 18,149, respectively (Fig. 2c). Furthermore, ELISpot assays confirmed the activity of RBD-specific B cells in the bone marrow and spleen following the three-dose regimen (Fig. 2d, e). Germinal center (GC) responses, essential for generating affinity-matured plasma cells and memory B cells, were also significantly enhanced in lymph nodes and spleens, with an increase in T follicular helper (Tfh) cells critical for long-lasting immunity (Fig. 2f, g). Consistent with previous findings that mRNA vaccines induce T helper type 1 (Th1) responses [14, 15], the JN.1-mRNA vaccine promoted a robust Th1-skewed cellular immune response. A significant proportion of CD4^+^ and CD8^+^ T cells produced Th1-associated cytokines, including IFN-γ, TNF-α, and IL-2 (Fig. 3a-c) [15, 25]. This Th1 bias was further validated by detecting IFN-γ^+^ secreting lymphocytes in the spleen via ELISpot analysis (Fig. 3d). In summary, the JN.1-mRNA vaccine demonstrated its potential to confer strong protection against COVID-19 by inducing robust neutralizing antibodies, a potent Th1-dominated cellular immune response, and favorable cytokine profiles. These findings highlight the vaccine's capacity to provide substantial protection against SARS-CoV-2 and its variants.

### The JN.1 mRNA vaccine provides long-term immune protection

**Fig. 4:**
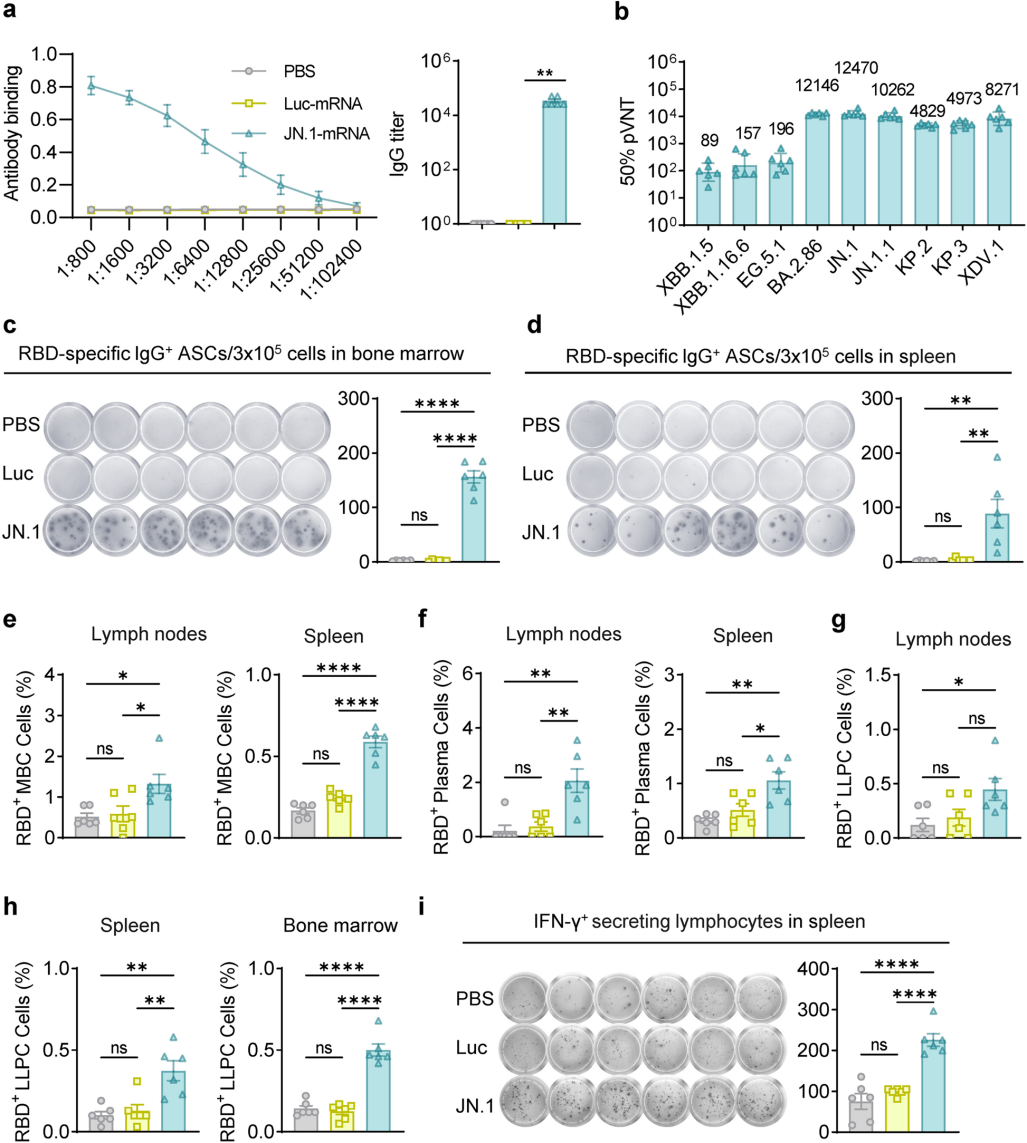
The JN.1 mRNA vaccine induced durable humoral and cellular immune responses. **a** The levels of RBD-specific IgG titers in serum, 6 months after the third immunization (*n* = 6). **b** Neutralizing antibody levels in serum at the same time point. **c**-**d** ELISpot images (left) and spot counts (right) of RBD-specific IgG^+^ ASCs in bone marrow (**c**) and spleen (**d**). **e**–**f** The percentage of RBD-specific MBCs (**e**), plasma cells (**f**) in lymph nodes (left) and spleen (right), 6 months after the third vaccination. **g**-**h** The percentage of RBD-specific LLPCs in lymph nodes (**g**) spleen (**h**, left), and bone marrow (**h**, right). **i** ELISpot images (left) and spot counts (right) of IFN-γ-secreting T cells in the spleen 6 months after the third vaccination. Statistical comparisons were performed using one-way ANOVA. *P < 0.05 was considered statistically significant, **P < 0.01, and ***P < 0.001 were considered highly significant

To evaluate the long-term immune protection provided by the JN.1-mRNA vaccine, serum and tissue samples were collected from mice six months after receiving the third dose. Despite a moderate decline in RBD-specific IgG titers and neutralizing antibody levels, these responses remained comparable, demonstrating sustained immunity over time (Fig. 4a, b). Notably, the GMTs for neutralizing BA.2.86, JN.1, JN.1.1, KP.2, KP.3 and XDV.1 pseudoviruses remained high, reflecting the continued effectiveness of the immune response (Fig. 4b). Additionally, JN.1-specific B cells were still detectable in the spleen and bone marrow, as well as RBD_JN.1_-specific memory B cells and plasma cells in the lymph nodes and spleen (Fig. 4c-f). The rising presence of long-lived plasma cells (LLPCs) in the lymph node, spleen and bone marrow crucial for maintaining long-term humoral immunity [26], further supports the durability of the immune protection six months post-vaccination (Fig. 4g, h). Moreover, IFN-γ-secreting cells, indicative of a robust cellular immune response, were detected in the spleen, highlighting the vaccine’s ability to elicit comprehensive immune protection (Fig. 4i). In summary, these findings confirm that the JN.1 mRNA vaccine induces enduring immunity through both humoral and cellular mechanisms, providing long-term protection against SARS-CoV-2 and its variants.

### Heterologous prime-boost vaccination induced stronger humoral immune response

**Fig. 5:**
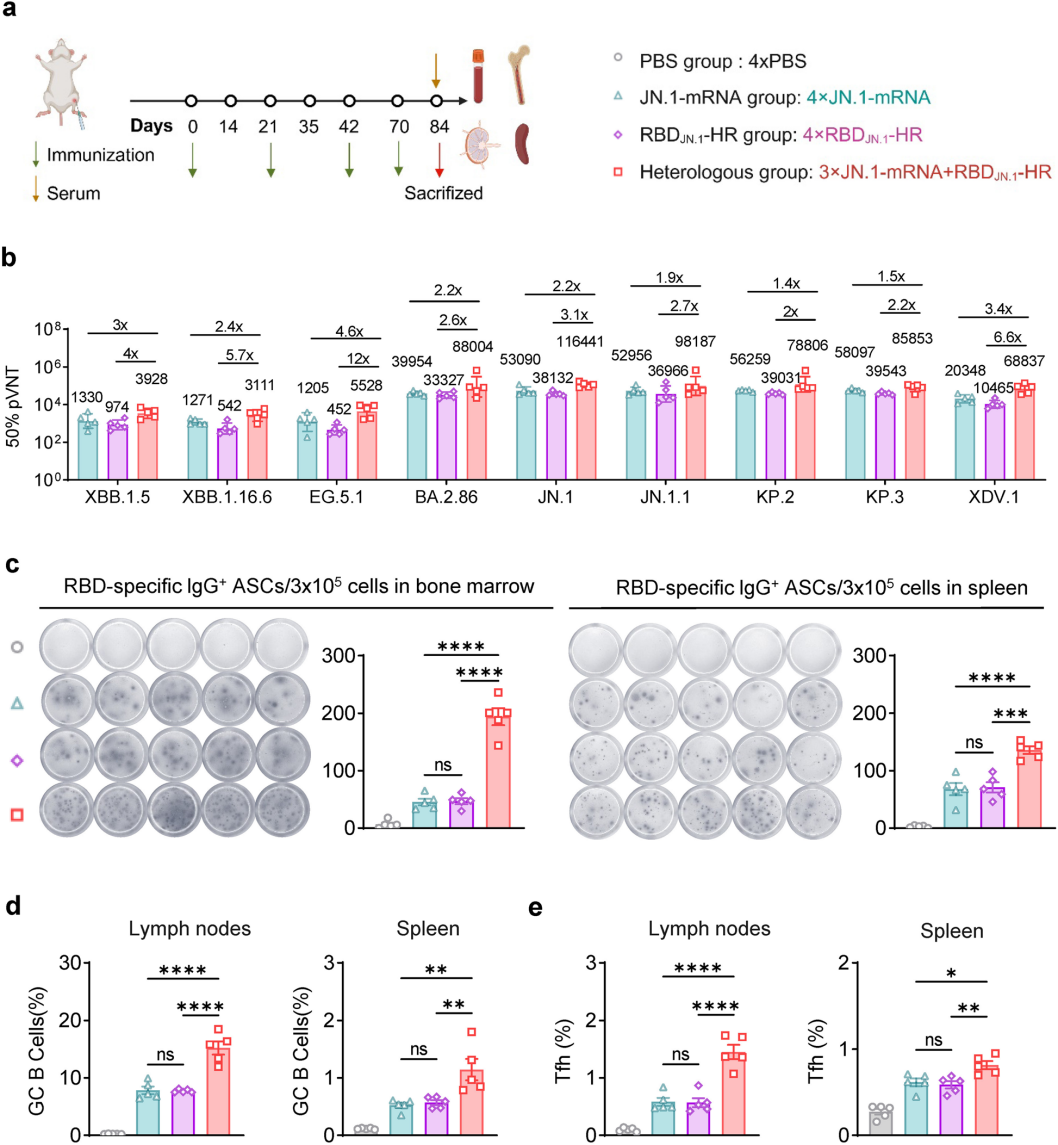
The RBD-HR protein booster elicits stronger humoral immunity than the mRNA booster. **a** Schematic diagram of the heterologous immunization, sample collection and groups (*n* = 5). **b** The detection of neutralizing antibodies. **c** ELISpot assays highlighted RBD-specific IgG^+^ ASCs in the bone marrow (left) and spleen (right) after boost vaccine. **d**-**e** The percentage of GC B cells (**d**) and Tfh cells (**e**) in lymph nodes (left) and spleen (right). Statistical comparisons were performed using one-way ANOVA. *P < 0.05 was considered statistically significant, **P < 0.01, and ***P < 0.001 were considered highly significant

To evaluate the impact of heterologous vaccination on immune responses, we compared the immune effects of homologous versus heterologous prime-boost regimens. In this study, we developed a trimeric RBD_JN.1_-HR recombinant protein as the heterologous booster vaccine to compare the immune responses induced by homologous vaccination and heterologous vaccination [23]. The fourth dose of the heterologous RBD_JN.1_-HR protein vaccine was administrated one month after the third dose priming JN.1-mRNA vaccine. As control, mice were immunized with four doses of either the protein vaccine or the mRNA vaccine on the same schedule (Fig. 5a). Encouragingly, the heterologous group elicited significantly higher levels of neutralizing antibodies against BA.2.86 and its subvariants than both homologous groups (Fig. 5b). Similar trends were observed in RBD-specific IgG^+^ antibody-secreting B cells from the bone marrow and spleen (Fig. 5c). Additionally, the heterologous group exhibited higher levels of GC B cells and Tfh cells in the lymph nodes and spleen (Fig. 5d, e). Overall, the heterologous vaccination strategy resulted in a stronger humoral immune response than the homologous regimen, whether with the JN.1-mRNA or RBD_JN.1_-HR vaccine.

### Heterologous prime-boost vaccination induced stronger cellular immune response

**Fig. 6:**
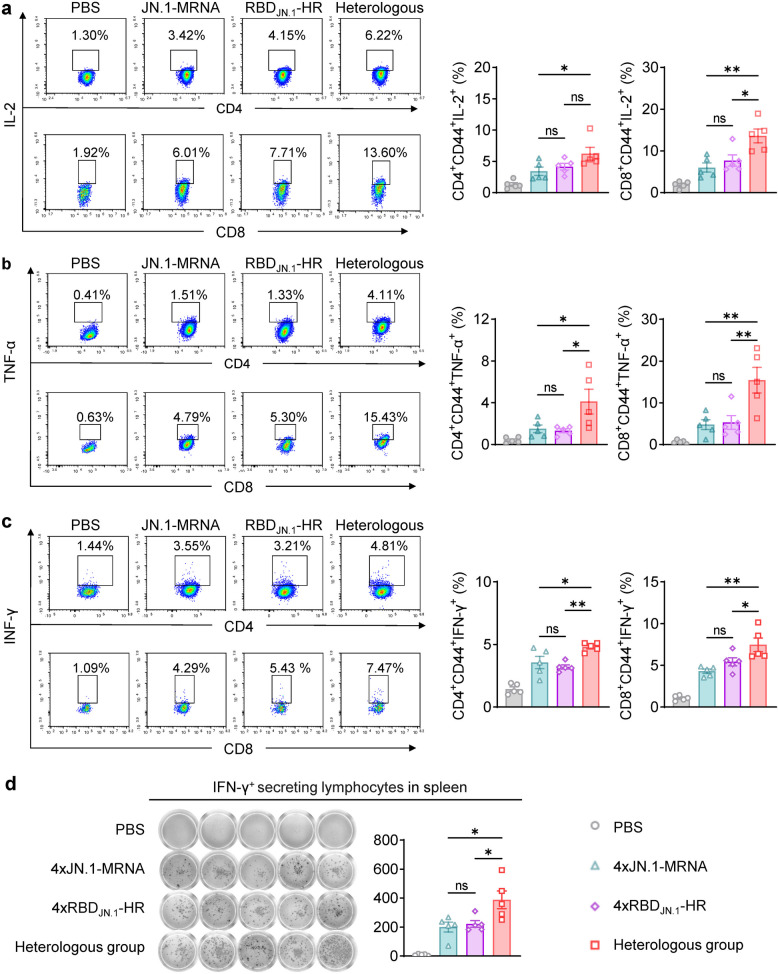
The RBD-HR protein booster elicits stronger Th1 immune responses than the mRNA booster Evaluation of Th1 immune responses 14 days after the booster vaccination (*n* = 5). Splenocytes were stimulated with JN.1 spike protein peptide pool and analyzed by flow cytometry for cytokine expression: IL-2 (**a**), TNF-α (**b**), IFN-γ (**c**). ELISpot images (left) and spot counts (right) of IFN-γ-secreting T cells in the spleen (**d**). Statistical comparisons were performed using one-way ANOVA. *P < 0.05 was considered statistically significant, **P < 0.01, and ***P < 0.001 were considered highly significant

In addition to humoral immunity, cellular immunity plays a critical role in combating viral infections including COVID-19 [27]. Cellular immunity provides a robust defense effect by eliminating virus-infected cells and coordinating adaptive immune responses. This T cell-mediated immunity often complements the neutralizing activity of antibodies and ensures a comprehensive response to infection including sustained protection. Our findings demonstrated that heterologous vaccination markedly enhanced Th1-type immune responses. This was evident from the significant upregulation of IL-2, TNF-α, and IFN-γ production by both CD4^+^ and CD8^+^ T cells (Fig. 6a-c). These cytokines are hallmarks of Th1 immunity and are critical for antiviral defense, as they activate macrophages, promote cytotoxic activity, and support the proliferation of effector T cells [28, 29]. Furthermore, IFN-γ-secreting lymphocytes were analyzed using an ELISpot assay to quantify antigen-specific T cell responses in the spleen. The robust presence of these cells indicated potent cellular immune activation, reflecting the vaccine's ability to stimulate durable T cell-mediated immunity (Fig. 6d). These results collectively highlight the advantages of heterologous vaccination strategies in inducing both humoral and cellular immunity, providing a synergistic approach to achieving effective and long-lasting protection against emerging variants of SARS-CoV-2.

## Discussion

In December 2023, the JN.1 was list as a variant of interest by WHO [30]. Compared to its predecessor BA.2.86, JN.1 contains an additional mutation in the spike protein, L455S, which enhances its infectivity and immune evasion abilities [3]. Vaccination continues to be a critical strategy in reducing hospitalization and mortality associated with COVID-19, but the efficacy of existing vaccines against new variants like JN.1 has become a concern. For example, the XBB.1.5 monovalent mRNA vaccine booster has shown significantly reduced efficacy against JN.1 when compared to its effectiveness against XBB.1.5 [31]. This underscores the critical need for the development of vaccines specifically targeting the JN.1 variant.

The principle behind the mRNA vaccine’s success lies in its rapid design and adaptability to new variants. The design of an mRNA vaccine encoding the full-length spike protein of JN.1, incorporating GSAS and 2P mutations to stabilize the spike protein, was based on the understanding that these modifications would enhance immune recognition and improve vaccine efficacy [32]. In our study, the JN.1-mRNA vaccine induced strong humoral and cellular immune responses in BALB/c mice, including high levels of RBD-specific IgG antibodies and cross-neutralizing antibodies against BA.2.86 and its sublineages (Fig. 2) (Figure S1). A single dose of the vaccine induced high titers of RBD-specific IgG antibodies, with subsequent doses further enhancing antibody levels (Fig. 2B). After three doses, immune sera contained high titers of cross-neutralizing antibodies against BA.2.86 and its sublineages (Fig. 2C). Robust Th1-type cellular immune responses were also detected in the spleen (Fig. 3). Remarkably, six months post-immunization, strong humoral and cellular immune responses were sustained in the blood, lymph nodes, spleen and bone marrow (Fig. 4). These results support the potential of mRNA-based vaccines to offer durable protection against evolving SARS-CoV-2 variants, which aligns with previous studies that showed long-lasting immune responses following mRNA vaccination [33–35].

Protein-based vaccines have several advantages, including great stability during storage and transportation, a well-established manufacturing process, and the ability to elicit strong immune responses [36]. Our group has successfully created a protein vaccine based on the RBD of the JN.1 variant (RBD_JN.1_-HR), which can self-assemble into a trimeric structure. To evaluate the immune-boosting potential of different vaccine strategies, we administered a RBD_JN.1_-HR protein vaccine booster to mice previously immunized with three doses of the JN.1-mRNA vaccine. Control groups received four doses of either the protein vaccine or mRNA vaccine. We observed that heterologous boosting with the RBD_JN.1_-HR protein vaccine after initial mRNA vaccination elicited a stronger immune response, both humoral and cellular, compared to homologous vaccine regimens (Figs. 5 and 6). This finding is consistent with previous studies that demonstrate the immunogenic advantages of heterologous prime-boost strategies [37]. This suggests that the heterologous boosting with different vaccine platforms may offer enhanced immunogenicity and could serve as a valuable complement or alternative to mRNA-based booster strategies.

While this study provides important insights, it is limited by the absence of direct evaluation of neutralizing antibodies against authentic SARS-CoV-2 in the serum of immunized mice, as well as the lack of viral challenge experiments to assess the protective efficacy. Additionally, considering the high prevalence of prior vaccination or infection among the population, a more effective way to assess the immune response induced by the JN.1-mRNA vaccine would be to administer it as a booster in individuals who have already received other primary vaccinations. This approach could offer valuable data on the potential of JN.1-mRNA as a supplementary strategy for enhancing immunity, particularly in the face of evolving viral variants.

In conclusion, we developed and evaluated an mRNA vaccine targeting the JN.1 variant of SARS-CoV-2. The JN.1-mRNA vaccine induced strong and long-lasting humoral and cellular immune responses in mice, including high levels of RBD-specific IgG antibodies and effective neutralizing antibodies against JN.1 lineages. While the immune responses induced by mRNA and protein vaccines were similar, sequential administration of both vaccines resulted in a more robust immune response compared to non-sequential vaccination. However, this study did not assess neutralizing antibodies against authentic SARS-CoV-2 or conduct viral challenge experiments, which limits direct conclusions about the vaccine's protective efficacy. Given the high prevalence of prior vaccination and infection, the JN.1-mRNA vaccine may prove particularly effective as a booster for individuals who have already been vaccinated against COVID-19.

## Material and methods

### Cell culture

The 293 T/ACE2 cell [38] were grown in Dulbecco’s modified Eagle’s medium (DMEM, Gibco, USA) supplemented with 10% heat-inactivated fetal bovine serum (FBS, Gibco) and 1% penicillin–streptomycin. The cells were maintained in a 37 ℃ incubator.

### Animals and immunization schedule

Healthy female BALB/c mice (6–8 weeks, 18–20 g) were ordered from Beijing Vital River Laboratory Animal Technology Co., Ltd. (Beijing, China) and maintained in the animal facility of the State Key Laboratory of Biotherapy, Sichuan University (Chengdu, Sichuan, China).

For the immunogenicity study of JN.1-mRNA, three groups of BALB/c mice (*n* = 11 per group) were injected 100 μl of PBS, Luc-mRNA (10 μg) or JN.1-mRNA (10 μg) by intramuscular (IM) route on days 0, 21, and 42, in the right leg. Blood samples were collected two weeks after each vaccination. 14 days after the last immunization, mice (*n* = 5 per group) were sacrificed, and peripheral blood, bone marrow, spleen, and lymph nodes were collected for analysis of JN.1-specific IgG, neutralizing antibody titers and the T-cell immune responses. Six months after the final immunization, mice (*n* = 6 per group) were sacrificed to measure the long-term immune response by collecting blood, bone, spleen, and lymph nodes.

To compared the effects of homologous and heterologous boosters, the BALB/c mice (*n* = 5 per group) were divided into four groups. The heterologous group was IM immunized with three doses of JN.1-mRNA (10 μg) vaccines on days 0, 21, and 42, followed by a heterologous booster of RBD_JN.1_-HR (10 μg) on day 70. Control groups received four doses of either PBS, RBD_JN.1_-HR (10 μg) or JN.1-mRNA (10 μg). Serum, bone marrow, spleen, and lymph nodes were collected 14 days after the final immunization.

### Tissues preparation

Blood samples were allowed to stand at room temperature for 4 h and centrifuged at 6000 rpm, 10 min at 4 °C. The serum was separated and stored at −80 °C for subsequent assays. Spleens were gently mashed using the 2 ml syringe plunger and filtered through a 70 μm cell strainer. Spleen lymphocytes were isolated using mouse lymphocyte separation medium (Dakewe Biotech Co., Ltd., China) and removing red blood cells. Similarly, lymph nodes and bone marrow were harvested from the right leg and processed to prepare single-cell lymphocyte suspensions. Those lymphocytes were analyzed by flow cytometry or ELISpot.

### JN.1-mRNA design and preparation

The JN.1 vaccine sequence is based on the full-length spike protein which contain the pre-fusion mutation (2P) and furin cleavage site mutation (GSAS). This spike sequence was cloned into our plasmid vector which included T7 promoter, 5′- and 3′-UTR, and 120-nucleotide polyA tail. Linearized DNA templates for the in vitro transcription (IVT) were prepared by digesting circular plasmid vector with *BspQ* I restriction enzyme (Hongene, Shanghai, China). T7 RNA polymerase (Hzymes Biotech, Wuhan, China), cap1 analog (Hzymes Biotech, Wuhan, China) and N1-methyl pseudouridine triphosphate (Glycogene, Wuhan, China) were used in the IVT reactions. mRNA was purified using oligo (dT) 30 magnetic particles (Vdobiotech, Suzhou, China) and stored at − 80 °C for future use.

### mRNA-LNP formulation and characterization

The mRNA was dissolved in 10 mM citrate buffer (pH 3.0) as the aqueous phase, while lipids including ALC-0315 (SINOPEG, Xiamen.China), DSPC (AVT, Shanghai,China), cholesterol (AVT, Shanghai,China) and DMG-PEG_2000_ (AVT, Shanghai,China) were dissolved in ethanol at a molar ratio of 50:10:38.5:1.5 as the organic phase. The mRNA-LNP formulation was prepared using a microfluidic device (Micro & Nano, Shanghai, China) to mix the aqueous and organic phases at a 3:1 flow ratio. The LNPs were then dialyzed using Tris buffer with sucrose in MWCO 6–8 kDa Pur-A-Lyzer dialysis tubes (Sigma-Aldrich, St. Louis, MO, USA) for 12 h to remove ethanol. mRNA-LNP formulations were then sterilized using filters (0.22 μm) and stored at −80° C until further use.

The particle size and zeta potential of mRNA-LNPs were analyzed with Zetasizer Pro (Malvern Panalytical, Malvern, UK). The morphology of the mRNA-LNPs was examined by transmission electron microscopy (TEM; H-600, Hitachi, Japan).

### Bioluminescence imaging

20 µg of Luc-mRNA formulation were injected intramuscularly into female BALB/c mice (*n* = 3 per group). After 6 h, mice were administrated intraperitoneally with 150 mg/kg luciferin (Aladdin, Shanghai, China). 10 min later, mice were euthanized, and images were captured using the IVIS Spectrum In Vivo Imaging System (PerkinElmer, Waltham, MA, USA).

### Enzyme-linked immunosorbent assays (ELISA)

To detect JN.1-specific IgG titers, ELISA plate (NUNC-MaxiSorp, Thermo Fisher Scien tific) was precoated with 100 μl of RBD_JN,1_-HR diluent at 1 μg/ml in carbonate buffer (50 mM, pH 9.6). After incubated overnight at 4 °C, the plate was washed three times with PBST (PBS with 0.1% Tween 20) and blocked with 1% BSA in PBST. Next, serially diluted sera were added to the washed plate and incubated at 37 °C for 2 h. Following wash step, horseradish peroxidase (HRP)‐conjugated anti‐mouse IgG antibodies (Invitrogen, 31,430) were diluted at 1:1000 in 1% BSA and added to the plate at 37℃. An hour later, washing plate at least five times, then TMB (Thermo Fisher Scientific, 34,029) was added to the plate. Following 10 min incubation in the dark, adding stop solution (Beyotime, P0215) and measuring the absorbance at 450 nm (630 nm as a reference) by a microplate reader (Spectramax ABS, Molecular Devices).

### ELISpot

ELISpot kits were used for detection of IFN-γ-secreting lymphocytes in the spleen (MABTECH, 3420-4APT-2) and IgG^+^ antibody-secreting cells (Millipore, MSIPS4W10) in the bone marrow and spleen. According to the instructions, the IgG^+^ ELISpot plate need precoated with RBD_JN.1_-HR (2 μg/mL) overnight at 4° C. Before adding lymphocytes suspensions, cleaning the IFN-γ ELISpot plate and coated IgG^+^ ELISpot plate for five time using PBS and then adding RPMI-1640 medium at 37 °C. 30 min later, the supernatant was removed, and 3 × 10^5^ lymphocytes were added to the plates for IFN-γ ELISpot, medium with JN.1 spike protein peptide pools was added.

After 20 h of incubation at 37 °C in 5% CO_2_, washing plates for five times to remove all the cells. For IFN-γ ELISpot, the 7-B6-1-biotin (1:1000) were added for 2 h at room temperature. After washing five times, streptavidin-ALP (1:1000) was added to the plates for 60 min. Flowing the washing step, BCIP/NBT-plus substrate solution was added to the well in the dark. According to the number of spots to stop the reaction using flowing Water. For IgG^+^ ELISpot, diluted HRP‐conjugated anti‐mouse IgG antibodies were added to the plate. After incubation for 2 h, washing the plate and TMB substrate was added. The plate can be rinsed with water and air-dry until spots appear. The number of spots were counted and scanned on an ELISpot reader.

### Pseudovirus neutralization assay

The pseudovirus neutralization assay was performed as previously described [39]. In brief, heat-inactivated sera samples were diluted by 1:3 or 1:9 and incubated in 96-well plates for 1 h at 37 °C with different pseudovirus (Genomeditech, China). Next, 2 × 10^4^ 293 T/ACE2 cells were added to each well. Two days later, the supernatant was removed, and luciferase substrate (Promega) was added to detect relative light units using a multimode microplate reader (PerkinElmer, USA).

### Flow cytometry

During the antibody staining, all the live lymphocytes cells were treated with PBS containing 1% BSA in ice. Tfh cells in spleen and lymph nodes were detected by the flowing directly labeled antibodies: B220- PE-Cy7 (BioLegend, 103,222), CD3-PerCP/Cyanine5.5 (BioLegend, 100,718), CD4-FITC (BioLegend, 100,406), CXCR5-APC (BioLegend, 145,506), PD-1-BV421 (BioLegend, 135,218). The GC B cells in spleen and lymph nodes were stained with B220- PE-Cy7 (BioLegend, 103,222), CD3-PerCP/Cyanine5.5 (BioLegend, 100,718), GL-7-APC (BioLegend, 144,618), CD95-FITC (BioLegend, 152,606). The gating strategy for Tfh and GC B were displayed in Supplementary Figure S1(A-B).

Six months after three doses of JN.1-mRNA, the lymphocytes cells in the bone marrow and spleen were initially incubated with biotinylated JN,1-RBD protein (ACROBiosystems, OBB1D-F001) at room temperature for 30 min. Flowing PBS washing, JN.1-specific memory B cells, Plasma Cells and LLPCs were detected through CD4-BV510 (BioLegend, 100,449), IgD-APC (BioLegend, 405,714), B220-PE/Cyanine5.5 (BioLegend, 103,209), CD38-PE/Cy7 (BioLegend, 102,718), GL-7-BV421 (BioLegend, 144,614), CD138-BV650 (BioLegend, 142,518), the PE Streptavidin (BioLegend, 405,204). The gating strategy for memory B cells, Plasma Cells and LLPCs were displayed in Supplementary Fig. S1 (C).

To determine the immune responses of JN.1-spike specific CD4^+^ and CD8^+^ T cells, 1.5 × 10^6^ splenic lymphocytes of each mouse were added to 12-well plates stimulated with 1 μg/ml peptide pools of JN.1 (Customer designed, GenScript). Cells were cultured in medium with 10% FBS, 100 μg/ml streptomycin, 100 U/ml penicillin, 1 mM pyruvate (all from Gibco, USA), 50 μM β-mercap-toethanol, and 20 U/ml IL-2 (all from Sigma-Aldrich, USA). After incubated 6 h, brefeldin A were added to the medium. The cells were harvested 12 h later and stained on ice for surface markers including Live/Dead-APC-Cy7 (Invitrogen™, L34976), CD3-PerCP/Cyanine5.5 (BioLegend, 100,718), CD4-BV421 (BioLegend, 100,438), CD8-FITC (BioLegend, 100,706), CD44-BV510 (BioLegend, 103,044) or CD44-PE (BioLegend, 103,024). Cells were then washed, fixed and permeabilized (BD Bioscience, 554,715) for 20 min. Then cells were washed by 1 × Perm/Wash™ and stained cytokines: IFN-γ- PE/Cyanine7 (BioLegend, 505,826), IL-2-APC (BioLegend, 503,810) and TNF-α-PE (BioLegend, 506,306). The gating strategy was displayed in Supplementary Fig. S2.

Flow cytometry data were analyzed by NovoCyte Flow Cytometer (ACEA Biosciences) and NovoExpress 1.4.1 software.

### Statistical analysis

All experiments were performed at least in duplicate unless otherwise specified, and all results are presented as the means ± SEM. Statistical analyses were conducted using Prism software (GraphPad Prism 8.0). Comparisons utilized One-way ANOVA followed by Tukey's multiple comparisons test. *P < 0.05; **P < 0.01; ***P < 0.001; ****P < 0.0001; ns, not significant.

## Supplementary Information

Supplementary Material 1

## Data Availability

All the data from the corresponding authors are available upon reasonable request.

## References

[1] Eshraghi R, Bahrami A, Karimi Houyeh M, Nasr Azadani M. JN.1 and the ongoing battle: unpacking the characteristics of a new dominant COVID-19 variant. Pathog Glob Health. 2024;118(6):453–8. 10.1080/20477724.2024.2369378. 38884317 10.1080/20477724.2024.2369378 PMC11441051

[2] Planas D, Staropoli I, Michel V, Lemoine F, Donati F, Prot M, et al. Distinct evolution of SARS-CoV-2 Omicron XBB and BA.2.86/JN.1 lineages combining increased fitness and antibody evasion. Nat Commun. 2024;15(1):2254. 10.1038/s41467-024-46490-7. 38480689 10.1038/s41467-024-46490-7 PMC10938001

[3] Yang S, Yu Y, Xu Y, Jian F, Song W, Yisimayi A, et al. Fast evolution of SARS-CoV-2 BA.2.86 to JN.1 under heavy immune pressure. Lancet Infect Dis. 2024;24(2):e70–2. 10.1016/s1473-3099(23)00744-2. 38109919 10.1016/S1473-3099(23)00744-2

[4] Kamble P, Daulatabad V, Singhal A, Ahmed ZS, Choubey A, Bhargava S, et al. JN.1 variant in enduring COVID-19 pandemic: is it a variety of interest (VoI) or variety of concern (VoC)? Horm Mol Biol Clin Investig. 2024;45(2):49–53. 10.1515/hmbci-2023-0088. 38622986 10.1515/hmbci-2023-0088

[5] Idris I, Adesola RO. Emergence and spread of JN.1 COVID-19 variant. Bull Natl Res Cent. 2024;48(1):27. 10.1186/s42269-024-01183-5.

[6] WHO. Statement on the antigen composition of COVID-19 vaccines. 2024. https://www.who.int/news/item/26-04-2024-statement-on-the-antigen-composition-of-covid-19-vaccines. Accessed April 26, 2024.

[7] FDA. Updated COVID-19 Vaccines for Use in the United States Beginning in Fall 2024. https://www.fda.gov/news-events/press-announcements/fda-roundup-june-7-2024. Accessed 7 June 2024.

[8] Szabó GT, Mahiny AJ, Vlatkovic I. COVID-19 mRNA vaccines: Platforms and current developments. Mol Ther. 2022;30(5):1850–68. 10.1016/j.ymthe.2022.02.016. 35189345 10.1016/j.ymthe.2022.02.016 PMC8856755

[9] Pardi N, Hogan MJ, Weissman D. Recent advances in mRNA vaccine technology. Curr Opin Immunol. 2020;65:14–20. 10.1016/j.coi.2020.01.008. 32244193 10.1016/j.coi.2020.01.008

[10] Bayani F, Hashkavaei NS, Arjmand S, Rezaei S, Uskoković V, Alijanianzadeh M, et al. An overview of the vaccine platforms to combat COVID-19 with a focus on the subunit vaccines. Prog Biophys Mol Biol. 2023;178:32–49. 10.1016/j.pbiomolbio.2023.02.004. 36801471 10.1016/j.pbiomolbio.2023.02.004 PMC9938630

[11] Zamani P, Mashreghi M, Rezazade Bazaz M, Zargari S, Alizadeh F, Dorrigiv M, et al. Characterization of stability, safety and immunogenicity of the mRNA lipid nanoparticle vaccine Iribovax® against COVID-19 in nonhuman primates. J Control Release. 2023;360:316–34. 10.1016/j.jconrel.2023.06.025. 37355212 10.1016/j.jconrel.2023.06.025

[12] Anand P, Stahel VP. The safety of Covid-19 mRNA vaccines: a review. Patient Saf Surg. 2021;15(1):20. 10.1186/s13037-021-00291-9. 33933145 10.1186/s13037-021-00291-9 PMC8087878

[13] Xu S, Yang K, Li R, Zhang L. mRNA Vaccine Era—Mechanisms, Drug Platform and Clinical Prospection. Int J Mol Sci. 2020;21(18):6582. 10.3390/ijms21186582. 10.3390/ijms21186582 PMC7554980 32916818

[14] Li M, Liu M, Song S, Zhao R, Xie Y, Liu J et al. Influenza a Neuraminidase-Based Bivalent mRNA Vaccine Induces Th1-Type Immune Response and Provides Protective Effects in Mice. Vaccines (Basel). 2024;12(3). 10.3390/vaccines12030300. 10.3390/vaccines12030300 PMC10974139 38543934

[15] Sahin U, Muik A, Derhovanessian E, Vogler I, Kranz LM, Vormehr M, et al. COVID-19 vaccine BNT162b1 elicits human antibody and TH1 T cell responses. Nature. 2020;586(7830):594–9. 10.1038/s41586-020-2814-7. 32998157 10.1038/s41586-020-2814-7

[16] Primorac D, Vrdoljak K, Brlek P, Pavelić E, Molnar V, Matišić V, et al. Adaptive Immune Responses and Immunity to SARS-CoV-2. Front Immunol. 2022;13: 848582. 10.3389/fimmu.2022.848582. 35603211 10.3389/fimmu.2022.848582 PMC9114812

[17] Schlake T, Thess A, Fotin-Mleczek M, Kallen K-J. Developing mRNA-vaccine technologies. RNA Biol. 2012;9(11):1319–30. 10.4161/rna.22269. 23064118 10.4161/rna.22269 PMC3597572

[18] Cagigi A, Loré K. Immune Responses Induced by mRNA Vaccination in Mice, Monkeys and Humans. Vaccines. 2021;9(1):61. 10.3390/vaccines9010061. 10.3390/vaccines9010061 PMC7831080 33477534

[19] Painter MM, Mathew D, Goel RR, Apostolidis SA, Pattekar A, Kuthuru O, et al. Rapid induction of antigen-specific CD4(+) T cells is associated with coordinated humoral and cellular immunity to SARS-CoV-2 mRNA vaccination. Immunity. 2021;54(9):2133–42.e3. 10.1016/j.immuni.2021.08.001. 34453880 10.1016/j.immuni.2021.08.001 PMC8361141

[20] Song Y, Wang J, Yang Z, He Q, Bao C, Xie Y, et al. Heterologous booster vaccination enhances antibody responses to SARS-CoV-2 by improving Tfh function and increasing B-cell clonotype SHM frequency. Front Immunol. 2024;15:1406138. 10.3389/fimmu.2024.1406138. 38975334 10.3389/fimmu.2024.1406138 PMC11224535

[21] Agrawal B. Heterologous Immunity: Role in Natural and Vaccine-Induced Resistance to Infections. Front Immunol. 2019;10:2631. 10.3389/fimmu.2019.02631. 31781118 10.3389/fimmu.2019.02631 PMC6856678

[22] Orlandi C, Stefanetti G, Barocci S, Buffi G, Diotallevi A, Rocchi E et al. Comparing Heterologous and Homologous COVID-19 Vaccination: A Longitudinal Study of Antibody Decay. Viruses. 2023;15(5). 10.3390/v15051162. 10.3390/v15051162 PMC10222288 37243247

[23] Yin WC, Xu YW, Xu PY, Cao XD, Wu CR, Gu CY, et al. Structures of the Omicron spike trimer with ACE2 and an anti-Omicron antibody. Science. 2022;375(6584):1048-+. 10.1126/science.abn8863. 35133176 10.1126/science.abn8863 PMC8939775

[24] Kalnin KV, Plitnik T, Kishko M, Zhang J, Zhang D, Beauvais A, et al. Immunogenicity and efficacy of mRNA COVID-19 vaccine MRT5500 in preclinical animal models. NPJ Vaccines. 2021;6(1):61. 10.1038/s41541-021-00324-5. 33875658 10.1038/s41541-021-00324-5 PMC8055913

[25] Yin X, Chen S, Eisenbarth SC. Dendritic Cell Regulation of T Helper Cells. Annu Rev Immunol. 2021;39:759–90. 10.1146/annurev-immunol-101819-025146. 33710920 10.1146/annurev-immunol-101819-025146

[26] Fooksman DR, Jing Z, Park R. New insights into the ontogeny, diversity, maturation and survival of long-lived plasma cells. Nat Rev Immunol. 2024;24(7):461–70. 10.1038/s41577-024-00991-0. 38332373 10.1038/s41577-024-00991-0

[27] Sette A, Sidney J, Crotty S. T Cell Responses to SARS-CoV-2. Annu Rev Immunol. 2023;41:343–73. 10.1146/annurev-immunol-101721-061120. 36750314 10.1146/annurev-immunol-101721-061120

[28] Chen J, Xiang X, Nie L, Guo X, Zhang F, Wen C, et al. The emerging role of Th1 cells in atherosclerosis and its implications for therapy. Front Immunol. 2022;13:1079668. 10.3389/fimmu.2022.1079668. 36685487 10.3389/fimmu.2022.1079668 PMC9849744

[29] West EE, Kolev M, Kemper C. Complement and the Regulation of T Cell Responses. Annu Rev Immunol. 2018;36:309–38. 10.1146/annurev-immunol-042617-053245. 29677470 10.1146/annurev-immunol-042617-053245 PMC7478175

[30] Looi MK. Covid-19: WHO adds JN.1 as new variant of interest. Bmj. 2023;383:2975. 10.1136/bmj.p2975. 38128957 10.1136/bmj.p2975

[31] Wang Q, Guo Y, Bowen A, Mellis IA, Valdez R, Gherasim C, et al. XBB.1.5 monovalent mRNA vaccine booster elicits robust neutralizing antibodies against XBB subvariants and JN.1. Cell Host Microbe. 2024;32(3):315–21.e3. 10.1016/j.chom.2024.01.014. 38377995 10.1016/j.chom.2024.01.014 PMC10948033

[32] Wang Y, Zhang Z, Luo J, Han X, Wei Y, Wei X. mRNA vaccine: a potential therapeutic strategy. Mol Cancer. 2021;20(1):33. 10.1186/s12943-021-01311-z. 33593376 10.1186/s12943-021-01311-z PMC7884263

[33] Srivastava K, Carreño JM, Gleason C, Monahan B, Singh G, Abbad A, et al. SARS-CoV-2-infection- and vaccine-induced antibody responses are long lasting with an initial waning phase followed by a stabilization phase. Immunity. 2024;57(3):587–99.e4. 10.1016/j.immuni.2024.01.017. 38395697 10.1016/j.immuni.2024.01.017 PMC11066813

[34] Han X, Alameh M-G, Butowska K, Knox JJ, Lundgreen K, Ghattas M, et al. Adjuvant lipidoid-substituted lipid nanoparticles augment the immunogenicity of SARS-CoV-2 mRNA vaccines. Nat Nanotechnol. 2023;18(9):1105–14. 10.1038/s41565-023-01404-4. 37365276 10.1038/s41565-023-01404-4

[35] Huang Q, Ji K, Tian S, Wang F, Huang B, Tong Z, et al. A single-dose mRNA vaccine provides a long-term protection for hACE2 transgenic mice from SARS-CoV-2. Nat Commun. 2021;12(1):776. 10.1038/s41467-021-21037-2. 33536425 10.1038/s41467-021-21037-2 PMC7858593

[36] Pollet J, Chen WH, Strych U. Recombinant protein vaccines, a proven approach against coronavirus pandemics. Adv Drug Deliv Rev. 2021;170:71–82. 10.1016/j.addr.2021.01.001. 33421475 10.1016/j.addr.2021.01.001 PMC7788321

[37] Zuo F, Abolhassani H, Du L, Piralla A, Bertoglio F, de Campos-Mata L, et al. Heterologous immunization with inactivated vaccine followed by mRNA-booster elicits strong immunity against SARS-CoV-2 Omicron variant. Nat Commun. 2022;13(1):2670. 10.1038/s41467-022-30340-5. 35562366 10.1038/s41467-022-30340-5 PMC9106736

[38] Yang J, Wang W, Chen Z, Lu S, Yang F, Bi Z, et al. Publisher Correction: A vaccine targeting the RBD of the S protein of SARS-CoV-2 induces protective immunity. Nature. 2021;590(7844):E23. 10.1038/s41586-020-03108-4. 33469221 10.1038/s41586-020-03108-4

[39] Yang J, Hong W, Lei H, He C, Lei W, Zhou Y, et al. Low levels of neutralizing antibodies against XBB Omicron subvariants after BA.5 infection. Signal Transduct Target Ther. 2023;8(1):252. 10.1038/s41392-023-01495-4. 37336889 10.1038/s41392-023-01495-4 PMC10279763

