# Correction: A promising mRNA vaccine derived from the JN.1 spike protein confers protective immunity against multiple emerged Omicron variants

**DOI:** 10.1186/s43556-025-00260-z

**Published:** 2025-03-17

**Authors:** Danyi Ao, Dandan Peng, Cai He, Chunjun Ye, Weiqi Hong, Xiya Huang, Yishan Lu, Jie Shi, Yu Zhang, Jian Liu, Xiawei Wei, Yuquan Wei

**Affiliations:** 1 Laboratory of Aging Research and Cancer Drug Target, State Key Laboratory of Biotherapy and Cancer Center, National Clinical Research Center for Geriatrics, West China Hospital, Sichuan University, No. 17, Block 3, Southern Renmin Road, Chengdu, 610041 Sichuan China; 2 WestVac Biopharma Co. Ltd., Chengdu, China

**Correction** **:** **Mol Biomed 6, 13 (2025)**

**https://doi.org/10.1186/s43556-025-00258-7**

Following publication of the original article [1], it is reported that the Competing interest section was incomplete due to a missing sentence. The section is corrected from:

**Competing interests**

This work was supported by the WestVac Biopharma Co. Ltd. Yuquan Wei and Xiawei Wei, who are also working at the WestVac Biopharma Co. Ltd. The remaining authors declare no conflict of interest.

To:

**Competing interests**

This work was supported by the WestVac Biopharma Co. Ltd. Yuquan Wei and Xiawei Wei, who are also working at the WestVac Biopharma Co. Ltd. Yuquan Wei is the Editors-in-Chief of Molecular Biomedicine, and he has not been involved in the process of the manuscript handling. The remaining authors declare no conflict of interest.

The original article [1] has been updated.
