# Supplementary material for: A promising mRNA vaccine derived from the JN.1 spike protein confers protective immunity against multiple emerged Omicron variants
Source: Mol Biomed. 2025 Mar 4;6:13. doi: 10.1186/s43556-025-00258-7 (PMC11880457; doi:10.1186/s43556-025-00258-7)
Supplement: Supplementary file 1 — Supplementary Material 1 [file 43556_2025_258_MOESM1_ESM.docx]

**Supplemental information**

**A promising mRNA vaccine derived from the JN.1 spike protein confers protective immunity against multiple emerged Omicron variants**

Danyi Ao^1, #^, Dandan Peng^1, #^, Cai He^1^, Chunjun Ye^1^, Weiqi Hong^1^, Xiya Huang^1^, Yishan Lu^1^, Jie Shi^1^, Yu Zhang ^1^, Jian Liu^1^, Xiawei Wei^1, 2*^, Yuquan Wei^1*^

# These authors contributed equally

* Correspondence and material requests should be addressed to Xiawei Wei, [xiaweiwei@scu.edu.cn](mailto:xiaweiwei@scu.edu.cn) and Yuquan Wei, [yuquanwei@scu.edu.cn](mailto:yuquanwei@scu.edu.cn).

^1^ Laboratory of Aging Research and Cancer Drug Target, State Key Laboratory of Biotherapy and Cancer Center, National Clinical Research Center for Geriatrics, West China Hospital, Sichuan University, No. 17, Block 3, Southern RenminRoad, Chengdu, Sichuan 610041, China.

^2^ WestVac Biopharma Co. Ltd., Chengdu, China.


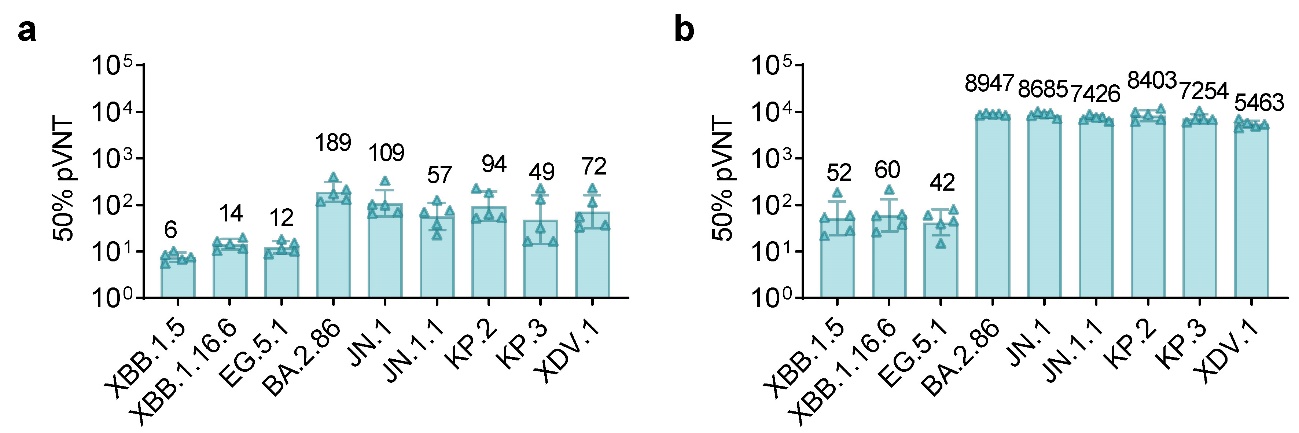


**Figure S1. Neutralizing antibody responses following JN.1-mRNA vaccination in mice.** (a) Neutralizing antibody titers measured 14 days after a single dose of the JN.1-mRNA vaccine (n=5). (b) Neutralizing antibody titers measured 14 days after a two-dose regimen of the JN.1-mRNA vaccine (n=5).


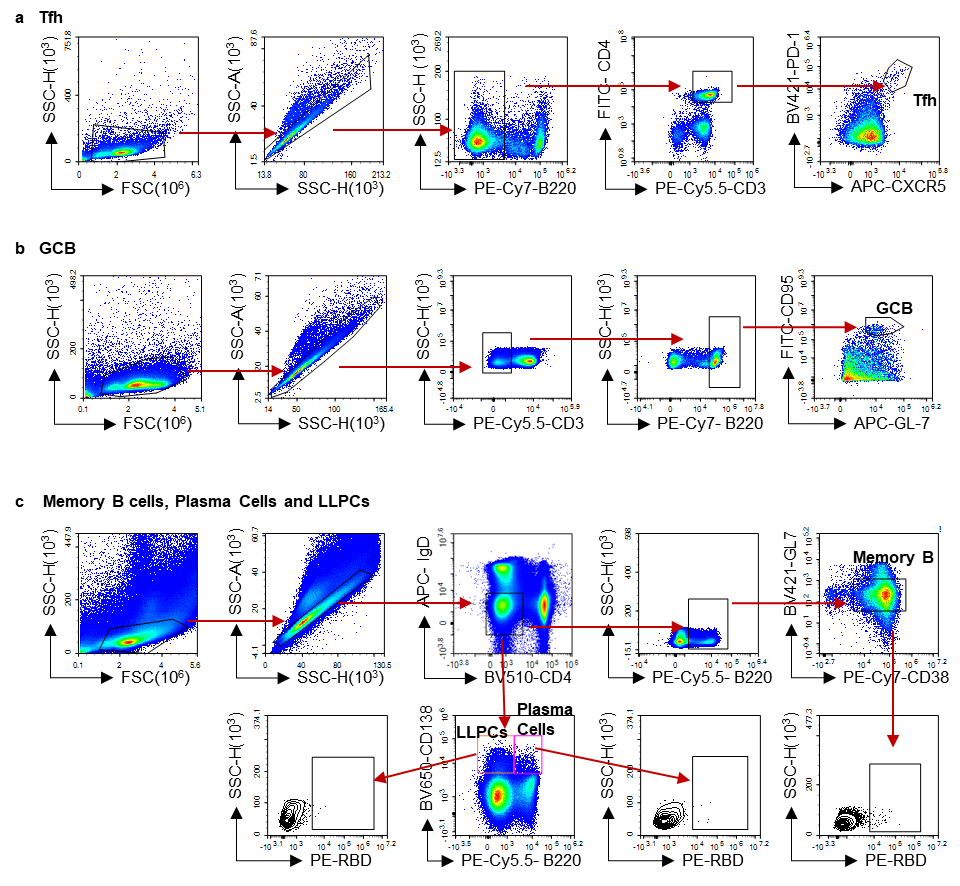


**Figure S2. The gating strategy for Tfh cells, GC B cells, Memory B cells, Plasma Cells and LLPCs.** (a) Gating strategy for Tfh cells (B220^-^CD3^+^CD4^+^PD-1^+^CXCR5^+^). (b) Gating strategy for GC B cells (CD3^-^B220^+^CD95^+^GL7^+^). (c) Gating strategy for RBS-specific Memory B cells (CD4^-^IgD^-^B220^+^CD38^+^GL7^-^ RBD^+^), Plasma Cells (CD4^-^IgD^-^B220^-^CD138^+^RBD^+^) and LLPCs (CD4^-^IgD^-^B220^+^CD138^+^RBD^+^).

**
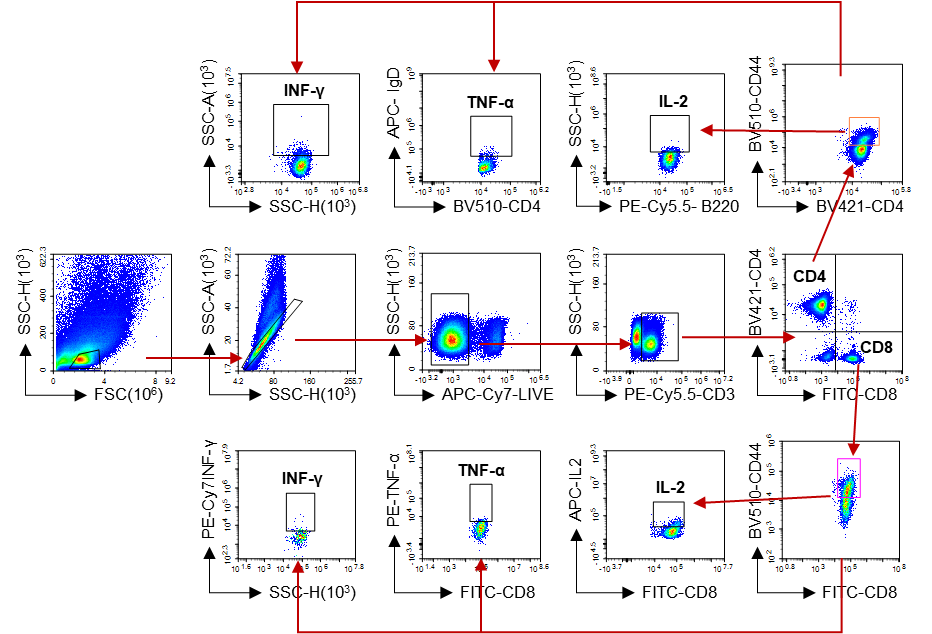
**

**Figure S3. The gating strategy for cellular cytokine-secreting T cells.** IFN-γ, IL-2 and TNF-α are secreted from CD3^-^CD4^+^CD44^+^ and CD3^-^CD8^+^CD44^+^ T cells.
